# Design and experiment of a combined peeling machine for water chestnut

**DOI:** 10.1038/s41598-023-28472-9

**Published:** 2023-02-10

**Authors:** Guozhong Zhang, Liming Chen, Zhou Guo, Haopeng Liu, Zhao Dong, Fang Liang

**Affiliations:** 1 College of Engineering, Huazhong Agricultural University, Wuhan, 430070 China; 2 Key Laboratory of Agricultural Equipment in Mid-lower Yangtze River, Ministry of Agriculture and Rural Affairs, Wuhan, 430070 China

**Keywords:** Engineering, Mechanical engineering

## Abstract

Water chestnut is a characteristic aquatic vegetable in China, and its demand for peeling fresh fruit is increasing rapidly. Aiming at the existing problems of high labor intensity and low efficiency of manual peeling, a combined water chestnut peeling machine was designed, which used a rotary knife to remove bud and root, and a differential friction belts to remove side peel. The performance of the peeling machine was tested with water chestnut from Xiaogan, Hubei Provence. Under the conditions of 200 g feeding mass and 10 r/min rotation speed, the single factor test was carried out with cutting speed as the influencing factor and the cutting rate of bud and root as the evaluation index. The results showed that the cutting rate of fresh fruit of water chestnut bud and root were 79.04% and 83.77% respectively when the cutting speed of rotary knife was 1.2 m/s. In the differential friction belts, high and low linear velocities were taken as the influencing factors, and the side peel removal rate was used as the evaluation index. The side peel removal rate was 84.93% at the high-speed linear velocity of 2.1 m/s and the low-speed linear velocity of 1.58 m/s. The performance of the whole machine was evaluated, and the results showed that the working loss of the combined water chestnut peeling machine was 43.03% and the comprehensive peeling rate was 77.43%, which reached the design requirements. This study can provide a reference for the research and development of water chestnut peeling device.

## Introduction

Water chestnut, also known as Eleocharis, is one of the important aquatic vegetables in China. It is widely planted in Hubei, Guangxi, Zhejiang, Hunan, and other places, with a total area of 50,000 hm^2^, and the annual output of fresh fruit of water chestnut is 600,000–800,000 tons^1,2^. At present, centering on the implementation of the national rural revitalization strategy and the needs of "one county, one product" county economic development, the planting of water chestnut in Guangxi, Hubei and other places is developing rapidly^3–5^. Fresh water chestnut after peeling can be used for the processing of candied fruit and canned fruit, which can achieve higher economic value^6,7^. However, water chestnut peeling is still mainly manually operated, with high labor intensity, low efficiency, and high production cost, which is difficult to meet the requirements of industrial development. The peeling technology has become one of the bottlenecks in the development of water chestnut industry.

Domestic and foreign scholars have studied common fruit and vegetable peeling technologies^8–11^, mainly including chemical peeling, steam peeling, mechanical peeling, etc.^12–15^. Steam peeling will make water chestnut cooked and lose their fresh flavor^16,17^, so this method is not feasible. Chemical peeling is soaked in lye, which is highly polluted by waste liquid and prone to lye residue and has an impact on food safety^18–20^. Mechanical peeling is the earliest and longest applied peeling method for fruit and vegetable, and it is also a more efficient and environmentally friendly method^21–23^. Chemical peeling and steam peeling can input a large number of target fruits at one time, so it can achieve high work efficiency. However, at present, the problem of pulp ripening or harmful liquid residues cannot be solved. Mechanical peeling will not cause potential safety and health hazards to fruits, and meets the food requirements. Therefore, the peeling industry generally focuses on machinery, such as Cao Chengmao, who designed a knife-cutting and rolling friction feeding bamboo shoots peeling machine, mechanized peeling of bamboo shoots^24^; Zeng Rong designed a multi-channel integrated shelling machine for fresh lotus seeds. The multi-channel profiling groove wheel was used to realize the single discharge of fresh lotus seeds, the circular cutting of internal and external cutters, and the separation of rolling shells and kernels, so as to realize the shelling of fresh lotus seeds^25^; Yu Guohong designed a flexible adaptive profiling sweet potato peeler based on the physical characteristics of sweet potatoes to achieve better sweet potato peeling performance^26^. Xu Xieqing designed a fresh lotus seed peeling machine based on the water jet peeling method to reduce manual work and improve peeling efficiency^27^.

The above peeling devices have realized the peeling function of agricultural materials in their respective fields, but cannot be applied to water chestnuts. Based on the status quo, the water chestnut mechanical peeling technology was studied in this paper, we designed a kind of combined water chestnut peeling machine, using the rotary knife to remove bud and root, differential friction belts to remove the side peel. Through the theoretical analysis of the main technical route such as blanking, positioning and transmitted through. The structure and parameter range of key components were determined, and the performance test was carried out, to provide a reference for the research and development of water chestnut peeling machine.

## Materials and methods

### Determination of the peeling scheme

**Figure 1:**
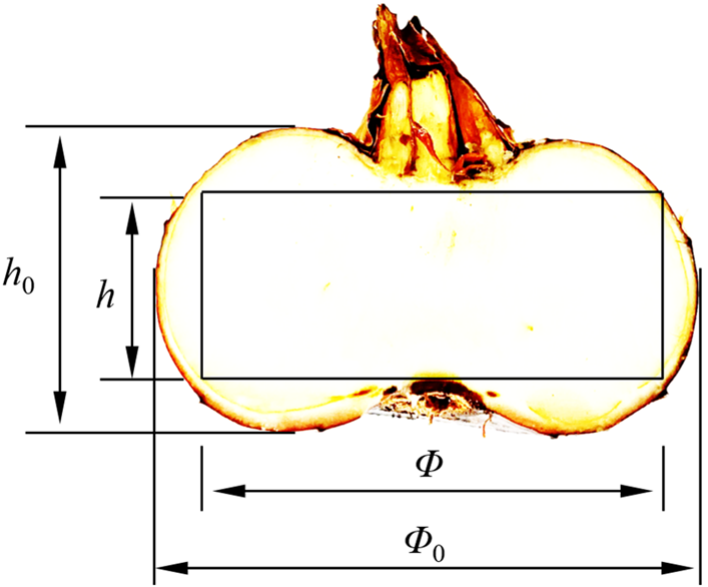
Water chestnut cross section; *h*_0_ is the total height of water chestnut; *h* is the maximum thickness of pulp after cutting bud and root; *Φ*_0_ is the transverse diameter; *Φ* is the maximum flesh diameter after removal of side peel.

The overall shape of water chestnut is similar to that of a lantern, but its shape is irregular. There is bud on the upper part, protruding upward and outward, and root on the bottom, with both bud and root sunken into the flesh (Fig. 1). Considering the shape of water chestnut, proposed the following technical scheme: the whole process of fruit peel can be divided into the removal of bud and root, and friction to remove side peel two phases in combination, to use a rotary knife center axis perpendicular to the water chestnut, at the same time close to bud and root, got removal of bud and root, then to remove side peel by friction way, to maximize the pulp of water chestnut. When bud and root were removed, the maximum thickness of pulp was retained according to the size *h* shown in Fig. 1. When rubbing the side peel, retain the maximum diameter pulp according to the size *φ* as shown in Fig. 1.

### Working principle

**Figure 2:**
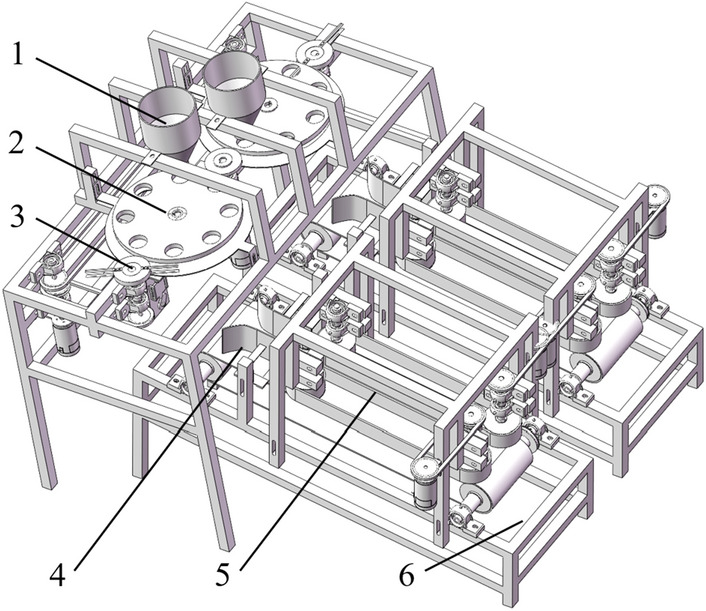
Three-dimensional structure of combined water chestnut peeling machine; (1) Blanking mechanism; (2) Positioning mechanism; (3) Cutting mechanism; (4) Transmission mechanism; (5) Friction mechanism; (6) Discharge mechanism.

The combined water chestnut peeling machine mainly consists of feeding, positioning, cutting, transmission, friction, unloading, etc. To improve the working efficiency, the machine adopts double-channel operation at the same time (Fig. 2).

**Figure 3:**
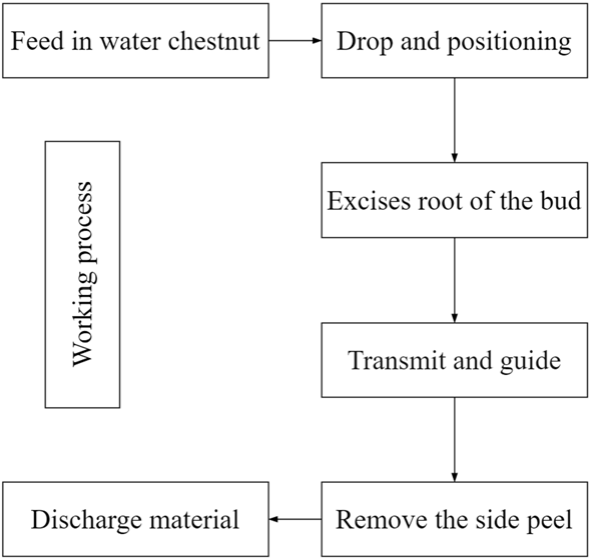
Working flow of combined water chestnut peeling machine.

When the peeling machine works, the water chestnut enters the feeding funnel by the feeding mechanism, and the discharging end of the funnel is brush. The water chestnut can be centralized and moved into the positioning hole of the positioning mechanism; the positioning mechanism is provided with an adjustable supporting plate, and the vibration source is installed below the supporting plate. When the water chestnut is rotated by the positioning mechanism, the supporting plate vibration makes the bud up and the root down. After alignment, the water chestnut moves to the cutting place driven by the positioning disk, and the rotary knife turns to remove the water chestnut bud and root at the same time; After the bud and root are removed, the water chestnut with side peel which is similar to drum enters the transmission mechanism, and then entered the conveying channel composed of differential friction belts and conveying flat belt under its guidance. Then, under the action of differential rubbing of friction belts on both sides, the side peel of water chestnut is removed, and the peeled water chestnut is uniformly recycled at the unloading place. The working process of the whole machine is shown in Fig. 3.

### Design of main working parts

#### Blanking and cutting device

**Figure 4:**
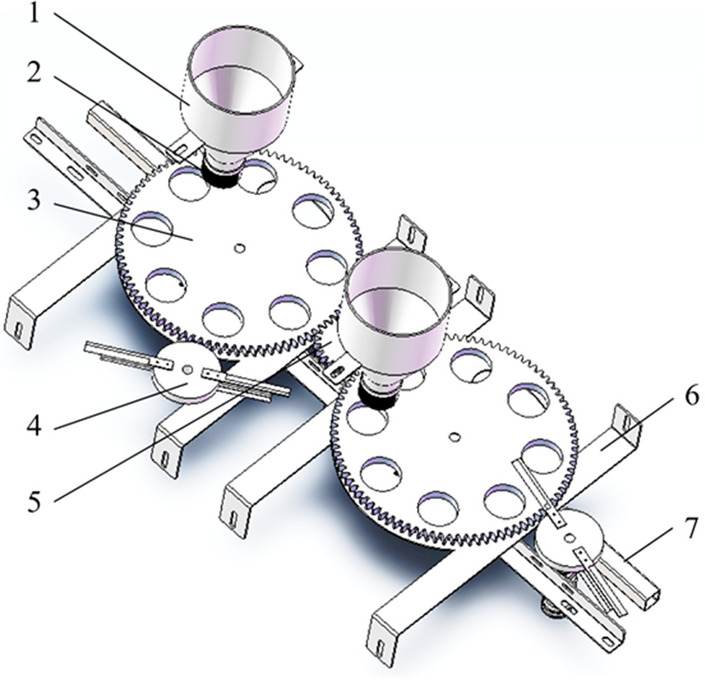
Blanking mechanism (1) Blanking hopper; (2) Brush; (3) Positioning disk; (4) Rotary knife; (5) Driving gear; (6) Supporting plate; (7) Supporting beam.

The positioning mechanism of the peeling machine is shown in Fig. 4. Its working process is as follows: Water chestnut falls to the upper surface of the positioning disk with the opening hole through the feeding hopper, and the positioning disk rotates under the drive of the motor. Under the action of the friction on the surface of the positioning disk and auxiliary pulling of the brush at the bottom of the hopper, the water chestnut falls into the positioning hole in turn. The diameter of the positioning hole is designed to ensure that only one water chestnut can enter, so the brush will send the redundant water chestnut fed by the subsequent hopper along the surface of the positioning disk and make it slide along the surface of the disk into the next positioning hole.

In addition, the brush can also adjust the posture of the water chestnut. According to the shape characteristics, there are only two ways to place the water chestnut: ① When the bud is upward and the bottom is downward, the water chestnut is relatively stable and will not be affected by the brush; ② When the bud is facing down, the water chestnut is extremely unstable. Under the effect of the brush, it will automatically turn over to place the bud facing up.

**Figure 5:**
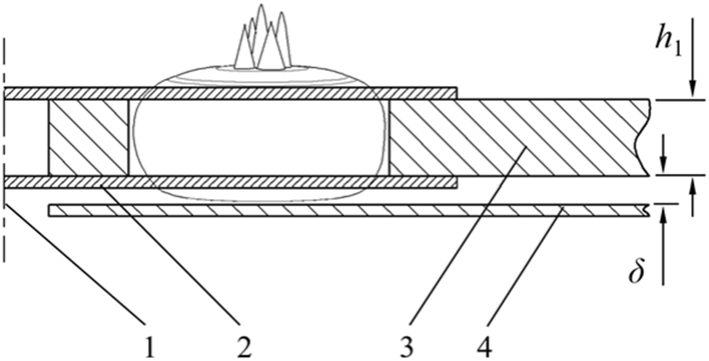
Cutting mechanism (1) Rotation center of the rotary knife; (2) Rotary knife; (3) Disk; (4) Supporting plate; *δ* is the gap between disk and supporting plate; *h*_1_ is positioning disk thickness.

The cutting mechanism is shown in Fig. 5. A stainless-steel supporting plate was arranged at the lower side of the positioning disk to hold the water chestnut falling into the positioning hole. There was a gap *δ* between the supporting plate and the disk, which can be set by adjusting the installation position of the supporting plate. When working, the lower knife rotated close to the lower surface of the positioning disk to remove the water chestnut root, while the upper knife rotated close to the upper surface of the positioning disk to remove the water chestnut bud part; It can be seen from Fig. 1 that both bud and root of water chestnut have depression, and bud (root) cutting rate can be controlled by adjusting gap *δ* and disk thickness *h*_1_. To give consideration to higher bud (root) cutting rate and flesh thickness *h*, this study focused on the average size of water chestnut, the gap was set at 3 mm, and disk thickness was set at 15 mm.

After removing the bud and root, the pulp entered the next procedure; bud and root left the disk area under the impact and friction of the knife, fell naturally, and were recycled uniformly. To compact the structure and reduce the external size of the whole machine, the diameter of the positioning disk was set as 380 mm, the number of positioning holes on the disk was 8, and the distance from the center of each positioning hole to the center of the rotating axis was 140 mm.

#### Analysis of transmission process of water chestnut

**Figure 6:**
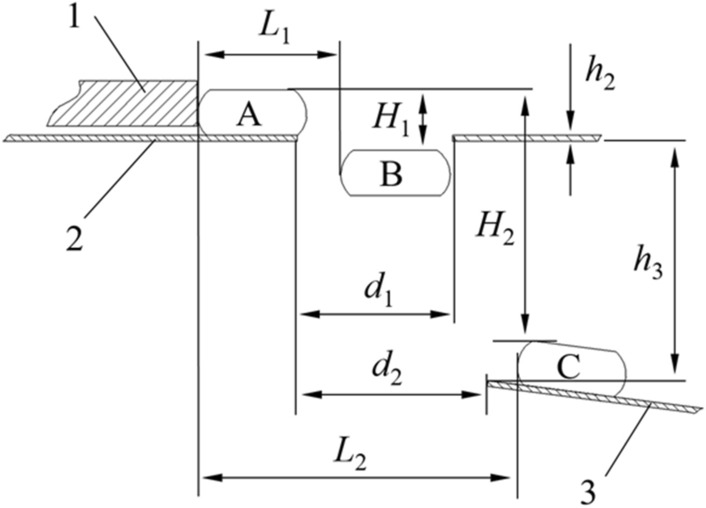
Analysis of the falling process of water chestnut (1) Positioning disk; (2) Supporting plate; (3) Conveyor; Water chestnut running track is A → B → C, A indicates the initial position of the water chestnut, B indicates that the water chestnut has just fallen into the opening, and C indicates that the water chestnut is in contact with the water chestnut conveyor; *h*_2_ is the thickness of the supporting plate, *h*_3_ is the distance between the supporting plate and the conveying mechanism, *L*_1_ is the distance from A to B, *L*_2_ is the distance from A to C, *H*_1_ is the height from A to B for the water chestnut, *H*_2_ is the falling height of water chestnut from A to C, *d*_1_ is the diameter of water chestnut blanking hole, *d*_2_ is the conveying mechanism and the spacing of blanking hole of supporting plate.

After the bud and root were cut off by the rotary knife, the water chestnut continued to move in a circular motion with the positioning disk, and when it moved to the opening of the supporting plate, it fell into the friction peeling device by gravity. To ensure that the water chestnut can fall smoothly from the opening to enter the next item of the friction mechanism, the process was analyzed. The movement model of the process is established as shown in Fig. 6.

The falling process of water chestnut is a flat throwing movement, and its horizontal velocity *v* is:

1 $$\left\{ {\begin{array}{*{20}l} {v = \omega r} \hfill \\ {\omega = \frac{\pi n}{{30}}} \hfill \\ \end{array} } \right.$$

The formula for calculating the size *d*_1_ at the opening of the vibration plate is as follows:

2 $$\left\{ {\begin{array}{*{20}l} {d_{1} \ge L_{1} + \frac{{\Phi_{0} }}{2}} \hfill \\ {L_{1} = vt_{1} } \hfill \\ {H_{1} = \frac{1}{2}gt_{1}^{2} } \hfill \\ {H_{1} = h + h_{2} } \hfill \\ \end{array} } \right.$$

The calculation formula for the installation size of water chestnut transfer mechanism is as follows:

3 $$\left\{ {\begin{array}{*{20}l} {d_{2} + \Phi_{0} \le L_{2} } \hfill \\ {L_{2} = vt_{2} } \hfill \\ {H_{2} = \frac{1}{2}gt_{2}^{2} } \hfill \\ {H_{2} = h + h_{2} + h_{3} } \hfill \\ \end{array} } \right.$$

where *v* is the linear velocity of the water chestnut in the positioning hole in a circular motion with the positioning disk, m/s; *ω* is the angular velocity of the disk, rad/s; *n* is the disk rotary speed, r/min; *r* is rotation radius of positioning hole, m; *t*_1_ is the time required for water chestnut to move from A to B, s; *t*_2_ is the time required for water chestnut to move from A to C.

The design range of disk rotary speed was 10–60 r/min; The thickness of the supporting plate had no direct influence on the working process, so the thickness was 2 mm which was the commonly used stainless steel plate; To make the whole machine structure compacted, the distance between the supporting plate and the transmission mechanism *h*_3_ was 100 mm; According to Eqs. (2) and (3), the dimension of the opening *d*_1_ ≥ 82 mm, and the distance between the installation position of the transmission mechanism and the left end of the opening of the support plate d2 ≤ 43 mm.

#### Profiling positioning block

After the water chestnut entered the positioning hole, to make it rotate steadily with the positioning disk in the positioning hole and prevent it from popping out from the hole during cutting, a profiling positioning block is designed. The structural design process was as follows.

**Figure 7:**
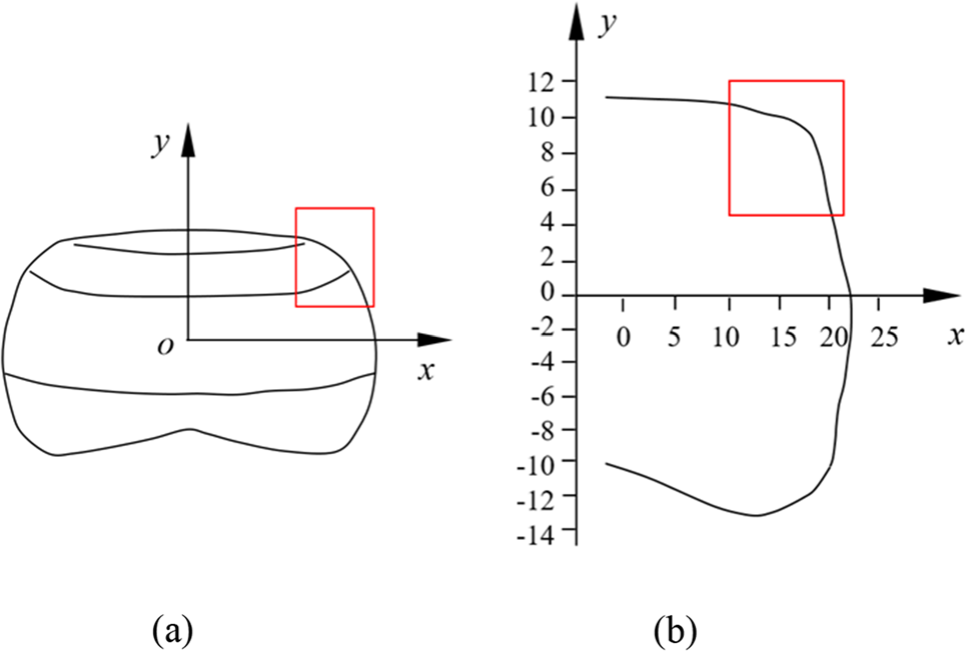
External contour fitting of water chestnut. (**a**) Establishment of the coordinate system of the water chestnut; (**b**) Fitting curve of external contour of water chestnut. The red rectangle is the final selection to establish the profiling positioning block.

Established the *x*-axis along the water chestnut midpoint horizontal transverse, and *y*-axis vertical longitudinal established (Fig. 7), the water chestnut dimension was measured along the longitudinal section contour, random measuring 20 sizes close to the average water chestnut, measuring the contour size, the water chestnut outer contour curve was obtained by MATLAB simulation data, established according to the outline curve contour locating piece, can make the water chestnut and its internal fit closely.

**Figure 8:**
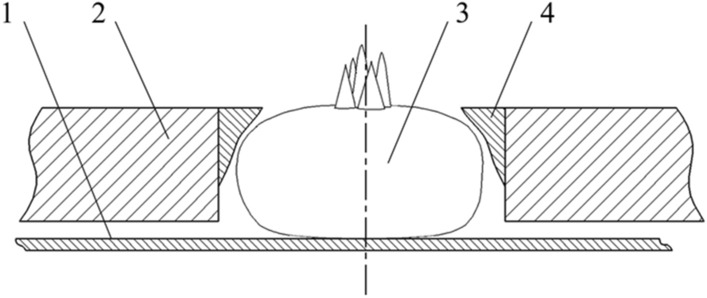
Schematic diagram of profiling fixture (1) Supporting plate; (2) Positioning disk; (3) Water chestnut; (4) Profiling block.

The contour parameters of the profiling positioning block were taken from a part of the contour curve of the water chestnut, as shown in the red rectangle in Fig. 7. Because of its complex curve form, the positioning profiling block was manufactured by 3D printing, and its outboard cylindrical surface was stuck and fixed on the inner wall of the positioning hole, as shown in Fig. 8, with the upper surface flushed with the positioning disk. When the water chestnut entered the positioning hole, its side fitted with the positioning block under the action of centrifugal force, compared with the cylindrical through-hole, the designed profiling positioning block not only increased the force contact area, but also facilitated the formation of stable supporting cutting.

#### Side peel removal device

**Figure 9:**
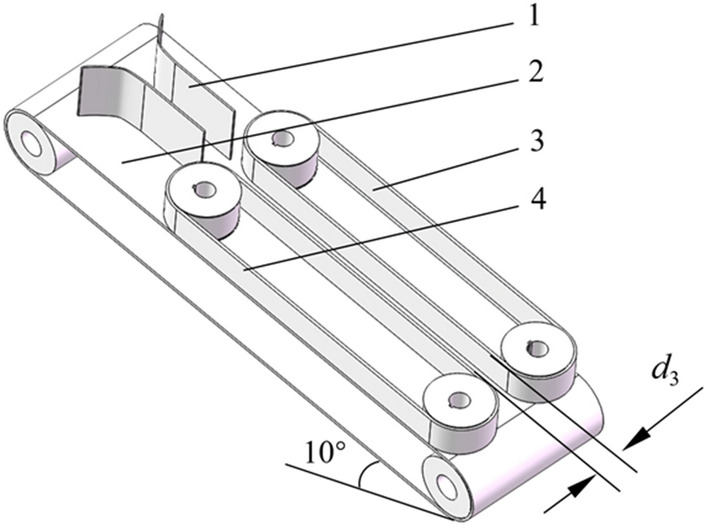
Schematic diagram of guiding device (1) Y-shaped port; (2) Conveyor belt; (3) Friction belt I; (4) Friction belt II; *d*_3_ is the inner distance of friction band.

After cutting the bud and root, the water chestnut fell into the Y-shaped feeding port at the blanking hole of the supporting plate, as shown in Fig. 9, the side peel removal process was continued. A conveyor belt is arranged below the Y-shaped feeding port, and the water chestnut was fed to the differential friction belts by the joint action of the guide plates on both sides. To ensure that water chestnut was better imported from the Y-shaped port, the conveyor belt is designed in an inclined state. After several tests, the inclined angle was set to 10°, and the effect of water chestnut entering the conveyor belt was the best.

**Figure 10:**
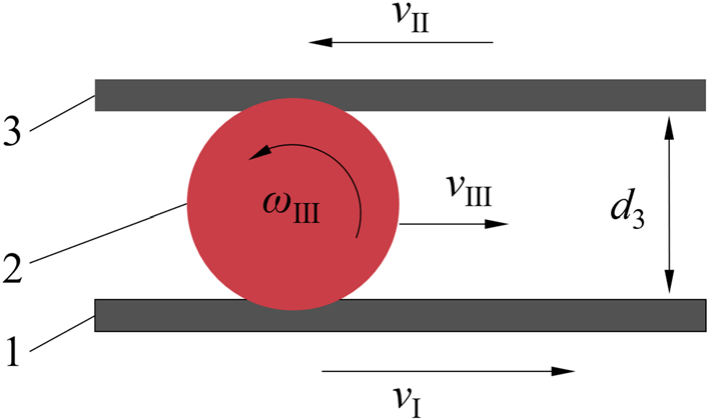
Principle of friction peeling (1) Friction belt I; (2) Water chestnut; (3) Friction belt II; *v*_I_ > *v*_I_; *v*_I_ is the velocity direction of the friction belt I; *v*_II_ is the velocity direction of the friction belt II; *v*_III_ is actual running direction of water chestnut; *ω*_III_ is actual angular velocity direction of water chestnut.

The friction belt spacing *d*_3_ can be adjusted by the installation position of the synchronous belt axle, and its size was the same as that *Φ* shown in Fig. 1, set the friction belt spacing *d*_3_ = 40 mm according to the size of the test object. The water chestnut was squeezed and rubbed by two differential friction belts and rotates forward in the channel to realize peeling all around. The principle was shown in Fig. 10.

The water chestnut rotated and advanced under the clamping effect of two differential friction belts, and its speed was *ω*_III_, the forward speed was *v*_III_; *v*_I_ and *v*_II_ were the linear velocity of high-speed belt and low-speed belt respectively, which were controlled by motor speed and adjustable; When the difference between the linear velocity of the differential friction belt *v*_I_ and *v*_II_ was larger, the forward speed of water chestnut *v*_III_ was larger; during the test, the linear speed of the differential belt was controlled by adjusting the motor speed, and then the forward speed of the water chestnut was changed to explore the corresponding working speed for the best peeling effect.

**Figure 11:**
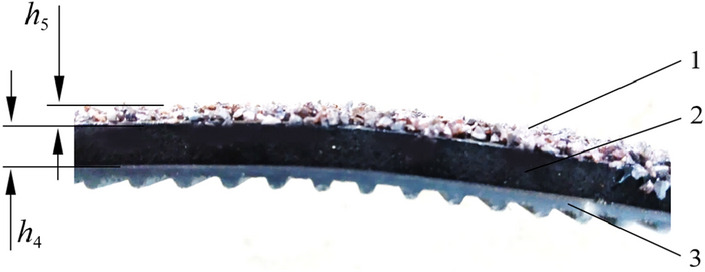
Schematic diagram of friction band (1) Friction particles; (2) Spongy layer; (3) Synchronous belt; *h*_4_ is the thickness of spongy layer; *h*_5_ is average grain size of sand.

The friction belt was developed by the team and consisted of a synchronous belt, sponge layer, and friction particles (Fig. 11). Synchronous belt and motor pulley connected, and plays a driving role; in combination with the external contour size of water chestnut, the sponge layer thickness *h*_4_ = 4 mm was set. By adding the sponge layer, it can play a role of buffering and decompression, effectively reduced the crushing damage of friction belt on water chestnut during peeling, reduced the loss of pulp, and ensured high peeling quality. Friction particles are composed of sand particles of different sizes. In order to ensure a better peeling effect, the average particle size of friction particles *h*_5_ = 2 mm, which is set based on the actual experiment, and the particle shape is polyhedral with sharp edges and corners, to quickly remove the side peel of water chestnut.

## Experimental design and analysis of results

### Test materials

The test material was the local traditional water chestnut variety widely cultivated in Xiaogan city, Hubei Province. The size and specifications were relatively consistent after classification and screening before the test. The average mass of single fruit was 29.03 g, the average maximum transverse diameter *Φ*_0_ was 44.99 mm, the average total pulp height *h*_0_ was 29.66 mm, and the average wet base moisture content was 83.32%. The test equipment included water content measuring instrument, electronic scale, speed calibration instrument, vernier caliper, box cutter, grid paper, marker pen, etc.

### Cutting test of bud and root

According to the pre-test, continuous feeding can be guaranteed when the single feeding was 200 g and the disk rotation speed was 10 r/min. Under these conditions, the knife rotation speed was taken as the influencing factor, the range of knife rotation speed *n* was set from 100 to 300 r/min, and the cutting rate of bud and root was taken as the evaluation index to carry out the single factor experiment on the optimal cutting speed of the peeling machine to cut root and bud.

**Figure 12:**
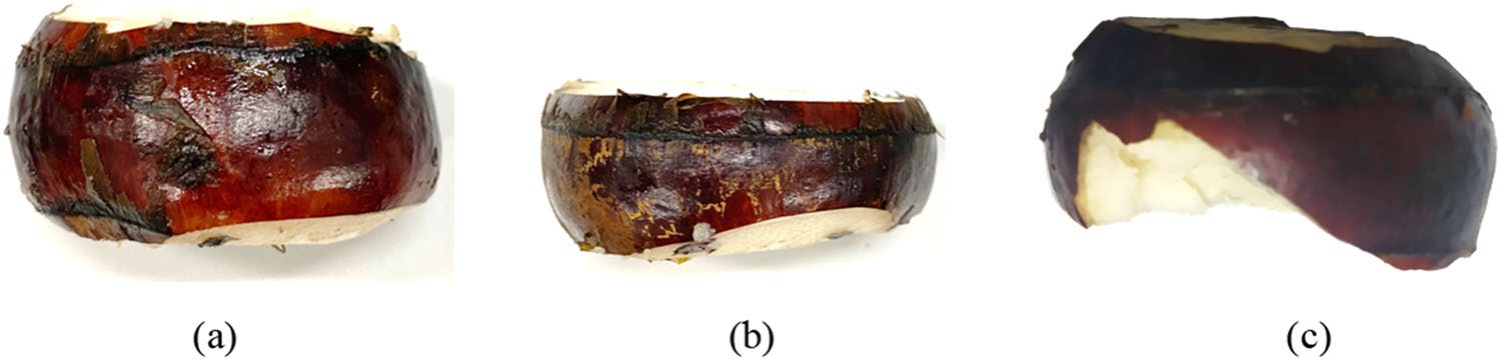
Water chestnut after repeated cutting (**a**) Normal cutting; (**b**) Repeated cutting 1; (**c**) Repeated cutting 2.

It was found that water chestnut would be repeatedly cut when cutting at high speed (Fig. 12). Repeated cutting results in the reduced thickness of water chestnut (Fig. 12b) or fragmentation of water chestnut (Fig. 12c), which increased additional peeling losses.

Analysis of the reason was that the cutting speed was large, the positioning disk did not send the water chestnut after cutting out of the cutting range in time, which led to secondary cutting. The main influencing factors were the knife speed, the rotation speed of the positioning disk, the center distance of the positioning hole, etc. In this study, the rotation speed of the knife and disk were taken as the influence factor.

The cutting speed *v*_1_ is the sum of the linear velocity of the positioning disk and the knife:

4 $$v_{1} = \omega \times r + \omega_{1} \times r_{1}$$

where *ω* is the angular velocity of the disk, rad/s; *r* is the rotation radius of the positioning hole, m; *ω*_1_ is the angular velocity of the knife, rad/s; *r*_1_ is the knife length, m.

The cutting rate of water chestnut bud (root) was calculated by the following formula:

5 $$y_{1} = \frac{{S_{a} - S_{r} }}{{S_{a} }} \times 100\%$$

where *y*_1_ is the cutting rate of bud (root), %; *S*_a_ is the section area after bud (root) excision, mm^2^; *S*_*r*_ is residual bud (root) area, mm^2^.

**Figure 13:**
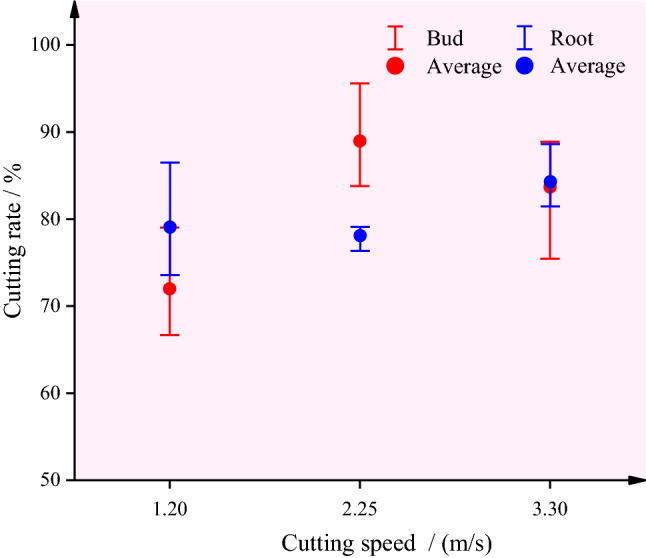
Result of bud and root resection.

The test result was shown in Fig. 13. Variance analysis was performed for the bud and root excision test of water chestnut as shown in Table 1. It can be seen that the cutting speed has no significant effect on the cutting of bud and root of water chestnut in the test range, and the difference in cutting rate is small with the increase of cutting speed.

**Table 1:** Variance analysis for rate of bud and root cutting.

Project	Source	*SS*	*df*	*MS*	*F* value
Bud	*SS*_A_	0.018109	2	0.0091	1.027304
	*SS*_e_	0.052883	6	0.0089	
	Total	0.070992	8		
Root	*SS*_A_	0.005440	2	0.00272	1.214261
	*SS*_e_	0.013439	6	0.00224	
	Total	0.018879	8		

Within the test range, according to the mean value analysis, when the cutting speed was 1.2 m/s, the removal effect of water chestnut bud and root was relatively the best.

### Removal test of side peel

Since there is no quality evaluation standard for water chestnut peeling at present, an evaluation method was proposed based on the current research situation. The removal rate of water chestnut side peel can be calculated by the following formula:

6 $$y_{2} = \frac{{S_{1} - S_{2} }}{{S_{1} }} \times 100\%$$

where *y*_2_ is the side peel removal rate, %; *S*_1_ is the cuticular area of water chestnut before removing side peel, mm^2^; *S*_2_ is the epidermal area of water chestnut after removing side peel, mm^2^.

In order to investigate the effect of working parameters on side peel removal by friction, a full factor experiment was carried out with water chestnut with bud and root removed as the object and the speed of differential belt as the influencing factor. The main function of the synchronous belt is to smoothly guide the water chestnut into the Y-shaped port and to ensure that water chestnut is always between the two belts during friction peeling, according to the actual test, when removing side peel, the water chestnut is mainly affected by friction between the two friction belts and is not in contact with the nether synchronous belt, so the speed of it had no effect, so did not take it as test factor.

**Figure 14:**
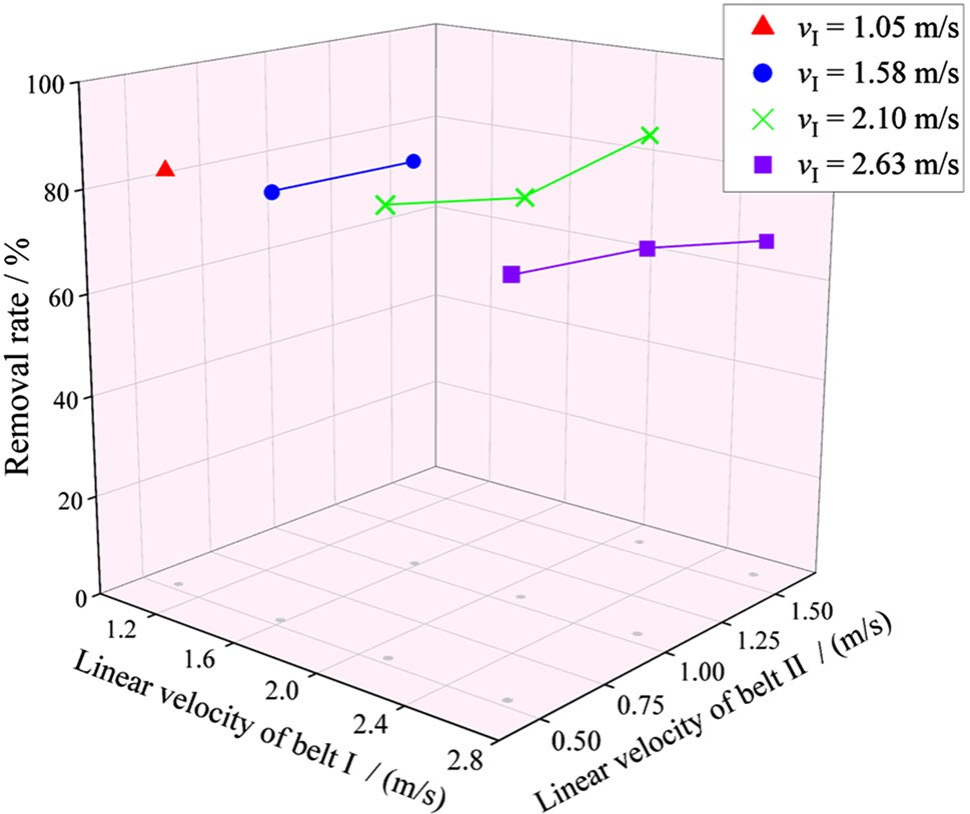
Removal rate of side peel of water chestnut.

The factors and levels of this test were shown in Table 2. According to the structure size, the rotation speed of the friction belt was converted to linear velocity. Belt I was the high-speed belt, and belt II was the low-speed belt. The test results were shown in Fig. 14.

**Table 2:** Side peel friction test factors and levels.

Level	Factor			
	Rotation speed of belt I/(r/min)	Linear velocity of belt I/(m/s)	Rotation speed of belt II/(r/min)	Linear velocity of belt II/(m/s)
1	200	1.05	100	0.53
2	300	1.58	200	1.05
3	400	2.10	300	1.58
4	500	2.63

**Figure 15:**
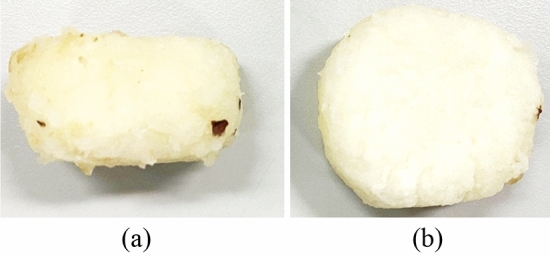
Water chestnut after friction peeling (**a**). Elevation view; (**b**). Top view.

The appearance of water chestnut after peeling was shown in Fig. 15. The results of variance analysis were shown in Table 3. The speed of the two friction belts had no significant influence on the peeling effect (*P* > 0.05), but with high-speed belt velocity increased, the side peel removal rate had a downward trend, mainly due to: When the side peel of water chestnut was removed between the differential channels of the two friction belts, the relative sliding distance between the side peel and the friction belts was always longer than the outer circumference of the water chestnut, and a certain amount of side peel can be removed. However, with the increase of the high-speed belt velocity, the relative sliding distance between the water chestnut and the friction belts decreased slightly, so the removal rate decreased. In the test range, according to the mean value analysis of test data, when *v*_I_ = 2.1 m/s and *v*_II_ = 1.58 m/s, the highest removal rate of water chestnut side peel was 84.93%.

**Table 3:** Analysis of variance.

Source	*SS*	*df*	*MS*	*F* value
Belt I	0.0173	3	0.0058	4.0500
Belt II	0.0015	2	0.0007	0.5222
Total	0.0188	5		

### Overall performance evaluation

**Figure 16:**
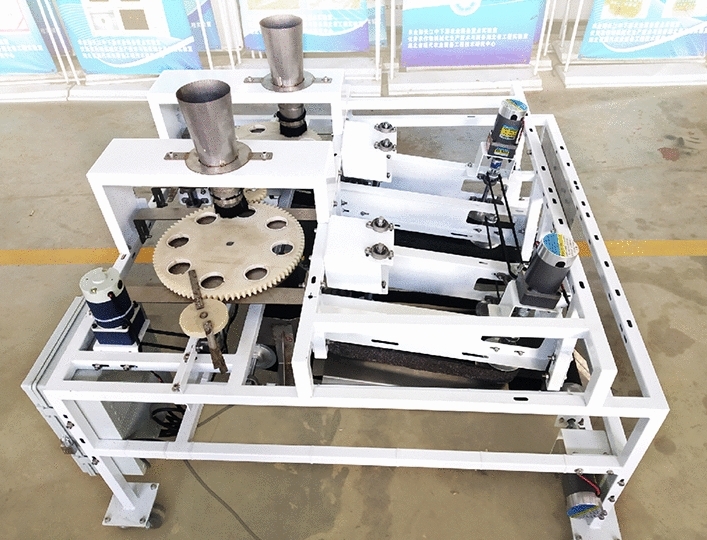
Combined peeling machine for water chestnut.

The combined water chestnut peeling machine was shown in Fig. 16. The parameters of the machine were adjusted according to the above tests, and the performance of the whole machine was evaluated under the optimal parameter combination of each project. The experiment was repeated 20 times, and the peeling loss rate and comprehensive peeling rate were obtained respectively.

The rate of peeling loss was calculated by the following formula:

7 $$y_{3} = \frac{{m_{1} - m_{2} }}{{m_{1} }} \times 100\%$$

where *y*_3_ is the loss rate, %; *m*_1_ is the total mass of water chestnut before peeling, g; *m*_2_ is the mass of water chestnut after peeling, g.

The comprehensive peeling rate was calculated by the following formula:

8 $$y_{4} = \frac{{y_{1} + y_{2} }}{2} \times 100\%$$

where *y*_4_ is the comprehensive peeling rate, %; *y*_1_ is the cutting rate of bud (root), %; *y*_2_ is the removal rate of side peel, %.

To prevent mass variation of water chestnut from water loss, an electronic scale was used to weigh the water chestnut immediately after the test. The results showed that under the optimal parameters, the working efficiency of the machine exceeded 6 kg/min, which was much higher than that of manual peeling; as for peeling quality, the peeling loss rate of the combined water chestnut peeling machine was 43.03%, and the comprehensive peeling rate was 77.43%. The peeling rate of the whole machine was different from that of the single bud, root, and side peel test. The main reason was that the peeling process of the whole machine was completed automatically, so there may be deviation in the transfer of water chestnut in different processes, which resulted in a poor effect.

Data showed that the peel of mature water chestnut accounted for a high proportion, about 20–25% of the total fruit mass^28–30^. Considering that the irregular shape of water chestnut will cause overcutting loss^31,32^ during mechanical peeling, combined with the actual investigation, the mass loss of manual peeling is about 40%. This peeling machine can realize the peeling loss of 43.03% and the comprehensive peeling rate of 77.43% respectively, achieving the design goal of initial replacement of the manual. However, the surface of water chestnut after peeling is rough and the perception is poor, which needs to be improved.

## Conclusion

In this study, a combined water chestnut peeling machine was designed, which used rotary knife to remove bud and root and differential friction belts group to remove side peel. The combined operation mode was adopted to remove bud and root and side peel of water chestnut in turn. Through the theoretical analysis of the process of blanking positioning and transmission, the structural form and parameter range of the device were determined.

Bench tests were carried out on the bud and root cutting and side peel friction of water chestnut. The results showed that when the cutting speed was 1.2 m/s, the cutting rate of bud and root of water chestnut could reach 79.04% and 83.77%; High speed belt *v*_I_ = 2.1 m/s, low speed belt *v*_II_ = 1.58 m/s, and the removal rate of water chestnut side peel was 84.93%. The performance of the whole machine was evaluated under the optimal parameter combination of each link. The results showed that the working efficiency of the machine can exceed 6 kg/min, and the peeling loss rate of the whole machine was 43.03%, and the comprehensive cleaning rate is 77.43%. The working indexes of the whole machine basically meet the design goal of replacing manual work. However, the peeled water chestnut has rough surface and high loss, which needs to be improved in the next study.

## Data Availability

All data generated or analyzed during this study are included in this published article and all data included in this study are available upon request by contact with the corresponding author.
